# Coiled-Coil Based Inclusion Bodies and Their Potential Applications

**DOI:** 10.3389/fbioe.2021.734068

**Published:** 2021-08-17

**Authors:** Marcos Gil-Garcia, Salvador Ventura

**Affiliations:** Institut de Biotecnologia i de Biomedicina and Departament de Bioquímica i Biologia Molecular, Universitat Autònoma de Barcelona, Bellaterra, Spain

**Keywords:** functional inclusion bodies, biomaterials, tags, coiled-coil, biotechnology, biomedicine, protein assembly

## Abstract

The production of recombinant proteins using microbial cell factories is frequently associated with the formation of inclusion bodies (IBs). These proteinaceous entities can be sometimes a reservoir of stable and active protein, might display good biocompatibility, and are produced efficiently and cost-effectively. Thus, these submicrometric particles are increasingly exploited as functional biomaterials for biotechnological and biomedical purposes. The fusion of aggregation-prone sequences to the target protein is a successful strategy to sequester soluble recombinant polypeptides into IBs. Traditionally, the use of these IB-tags results in the formation of amyloid-like scaffolds where the protein of interest is trapped. This amyloid conformation might compromise the protein’s activity and be potentially cytotoxic. One promising alternative to overcome these limitations exploits the coiled-coil fold, composed of two or more α-helices and widely used by nature to create supramolecular assemblies. In this review, we summarize the state-of-the-art of functional IBs technology, focusing on the coiled-coil-assembly strategy, describing its advantages and applications, delving into future developments and necessary improvements in the field.

## Introduction

The use of microorganisms as cell factories to produce biological products (as therapeutic proteins), often leads to the formation of insoluble protein deposits in their cytoplasm (Villaverde and Carrió, 2003). These protein deposits, commonly known as inclusion bodies (IBs), have been considered a significant bottleneck for obtaining high yields of well-folded and soluble protein since they were understood as reservoirs of inactive protein (Baneyx and Mujacic, 2004).

IBs are refractile and submicron protein nanoparticles (between 50–1,000 nm) that usually accumulate at the poles of the cell (Luo et al., 2006; Margreiter et al., 2008; García-Fruitós et al., 2009, García-Fruitós et al., 2012; Castellanos-Mendoza et al., 2014). These proteinaceous inclusions are mechanically stable in a wide range of temperatures and pHs, present a pseudo-spherical shape, and are mainly composed of the target protein (De Marco et al., 2019); although the composition and purity of the IBs significantly depends on the identity of the recombinant protein. Moreover, a significant fraction of the protein trapped in these IBs can be well-folded and functional, suggesting that these nanoparticles might be a source of active protein (Ventura and Villaverde, 2006). Due to their inherent stability, they can be easily purified by simple cell disruption methods, obtaining high yields of pure protein cost-effectively. Furthermore, purified IBs can be stored in lyophilized form or at −80°C for extended periods (García-Fruitós et al., 2009).

IBs usually present an amyloid-like architecture responsible for their excellent mechanical robustness, as corroborated by several biophysical techniques (Carrió et al., 2005; Morell et al., 2008; Wang et al., 2008). However, despite this amyloidogenic character and the intrinsic toxicity of amyloid oligomeric forms (Díaz-Caballero et al., 2020), these IBs are assumed to be non-toxic materials, and thanks to their submicrometric size and functional character, they have found biomedical and biotechnological applications (Villaverde et al., 2015).

The formation of insoluble IBs can be induced by fusing aggregation-prone peptide or protein tags to the globular protein of interest, irrespectively of its solubility. Typically, the tag provides the driving force for forming the intermolecular β-sheet contacts that sustain the IBs amyloid-like nanostructure. Different research groups have used coiled-coil domains as IB-inducing tags (IB-tags) with significant success in the last years, suggesting that these α-helix-based tags are a feasible alternative to the β-sheet-based ones (Jäger et al., 2020).

In this mini-review, we illustrate recent advances in the field of functional IBs, discussing their biotechnological and biomedical applications, with particular emphasis on the uses of the new class of coiled-coil IBs. We also describe the assembly of cell component-free artificial IBs.

## Protein Inclusion Bodies as an Unexpected Source of Active Protein

The development of recombinant DNA technologies has allowed producing a wide diversity of proteins using heterologous expression systems. This includes therapeutic proteins such as replacement enzymes, hormones, or antibodies (Sanchez-Garcia et al., 2016; Walsh, 2018).

Proteins function at concentrations at which they remain soluble, whereas exceeding their solubility limits results in their aggregation and inactivation, according to “the life on the edge” hypothesis (Tartaglia et al., 2007; Vecchi et al., 2020). Therefore, the proteins' cellular levels are tightly regulated, both in time and space, according to their intrinsic solubilities (de Groot and Ventura, 2010; Castillo et al., 2011; Sanchez de Groot et al., 2015). The production of such polypeptides recombinantly often surpasses several orders of magnitude these solubility limits, which together with the lack of post-translational modifications or the deregulation of the refolding machinery, trigger the occurrence of protein misfolding and aggregation events inside the cell. This unavoidable connection between intracellular protein concentration and aggregation constitutes one of the main limitations for producing recombinant proteins at preparative levels in the biotechnological and pharmaceutical industry, evoking a considerable loss of resources and time (Roberts, 2014). Therefore, a significant effort has been devoted to implementing strategies aimed to increase the yield of soluble and functional protein, such as the redesign of the intrinsic protein solubility (Gil-Garcia et al., 2018) or the fusion of solubility tags (Costa et al., 2014), among others. These approaches try to push the solubility of the target protein above its natural limit. Soluble and folded protein has been obtained from IBs by denaturing-renaturing procedures, but the process is cost-ineffective and needs to be tailored for the protein of interest (Singhvi et al., 2020).

Traditionally understood as undesired byproducts of protein production processes, IBs have been ignored and avoided for decades. Two independent studies published in the 80–90s reported biological activity in the IBs formed by two different enzymes (β-galactosidase and endoglucanase D) (Worrall and Goss, 1989; Tokatlidis et al., 1991). However, they were considered anecdotic, and only when, in 2005, two additional studies recapitulated these data, our perception of IBs molecular properties changed dramatically. Jevsevar and coworkers obtained IBs of the human granulocyte-colony stimulating factor from where the functional protein could be easily isolated without the need for denaturing-renaturing steps (Jevsevar et al., 2005). In parallel, García-Fruitós and coworkers demonstrated that the IBs formed by two fluorescent proteins (blue and green fluorescent proteins) and two enzymes (human dihydrofolate reductase (DHFR) and *E. coli* β-galactosidase) were functional (García-Fruitós et al., 2005).

The awareness that IBs were functional particles, together with their easy and cost-effective production and the remarkable mechanical properties of such nanoparticles, paved the way for creating rationally designed IBs with a wide variety of functionalities.

## The Use of IB-Tags as a Smart Strategy for Obtaining Cost-Effective and Ready-To-Use Functional IBs

The formation of IBs involves the establishment of homotypic intermolecular interactions, a process that is strongly dependent on the microenvironment. In this way, it has been demonstrated that different cell culture variables such as the pH of the solution (Castellanos-Mendoza et al., 2014; Calcines-Cruz et al., 2018), the production temperature (de Groot and Ventura, 2006; Peternel et al., 2008; Seras-Franzoso et al., 2014), the time of induction (Margreiter et al., 2008) or the concentration of inducer agent (Luo et al., 2006), influence not only the kinetics of IBs formation but also the size, stability, and activity of these nanoparticles (De Marco et al., 2019).

Strange as it may seem, it is as challenging to produce an aggregation-prone protein in a soluble conformation as it is to force a soluble protein to form IBs efficiently, regardless of the culture conditions. In this latter case, the fusion of an aggregation-prone sequence to the target protein, commonly known as IB-tag, may solve the problem and incorporate the protein of interest into IBs.

In the last years, different IB-tags, consisting of small artificial peptides or large protein domains, have been exploited to create functional IBs (Krauss et al., 2017; Jäger et al., 2020).

A large group of such tags corresponds to small artificial peptides such as the β-sheet forming ELK16 or the hydrophobic GFIL8 peptides, that have been exploited for forming catalytically active IBs composed of oxidases, hydrolases, and oxidoreductases (Wu et al., 2011; Wang et al., 2015).

Apart from these small IB-tags, other aggregation-tags involve natural protein domains such as the viral capsid protein (VP1) of the foot-and-mouth disease virus and the variant F19D of the amyloid β-peptide (Aβ42). The VP1 domain has been exploited for obtaining functional IBs in the yeast *Pichia pastoris*, constituting an alternative for producing IBs of proteins needing post-translational modifications (Rueda et al., 2016). In the case of Aβ42, it has been used by our group to model amyloid aggregation and identifying chemical modulators of this deleterious reaction (De Groot et al., 2006; Dasari et al., 2011; Villar-Piqué et al., 2012).

Alternative aggregation-prone domains for obtaining catalytic IBs are the cellulose-binding domain (CBD) of different organisms such as *Clostridium cellulovorans* and *Cellulomonas fimi* (Nahalka and Nidetzky, 2007; Choi et al., 2011), a variant of the maltose-binding protein (MBP), known as MalE31, from *E. coli* (Arié et al., 2006) and the pyruvate oxidase (PoxB) from *Paenibacillus polymyxa* (Park et al., 2012). These IB-tags have been used to produce catalytic IBs composed of amylases, alkaline phosphatases, and β-lactamases, among other enzymes. Interestingly, the IBs promoted by MalE31 are located at the periplasm of the *E. coli* cell, facilitating their isolation. Furthermore, the IBs formed by PoxB present an intrinsic enzymatic activity, allowing to obtain IBs with simultaneous and different catalytic activities depending on the appended enzyme.

Finally, the signal sequence of *E. coli* TorA (ssTorA) has been successfully used to promote the accumulation into IBs of two highly soluble proteins, the MBP and the thioredoxin-1 (TrxA), usually exploited as solubility-enhancing fusion tags (Jong et al., 2017), demonstrating the pro-aggregational potency of this IB-tag. Moreover, a subsequent study has demonstrated that the IBs formed by the fusion of ssTorA to ovalbumin (OVA)-derived epitopes can act as an antigenic vaccine formulation for T cell response (Schetters et al., 2020). Mutagenesis-screening of the ssTorA sequence has allowed the identification of improved versions of the tag with enhanced IB-formation properties (Jong et al., 2019).

## Coiled-Coil Domains as Scaffolds for the Creation of Highly Functional IBs

**FIGURE 1:**
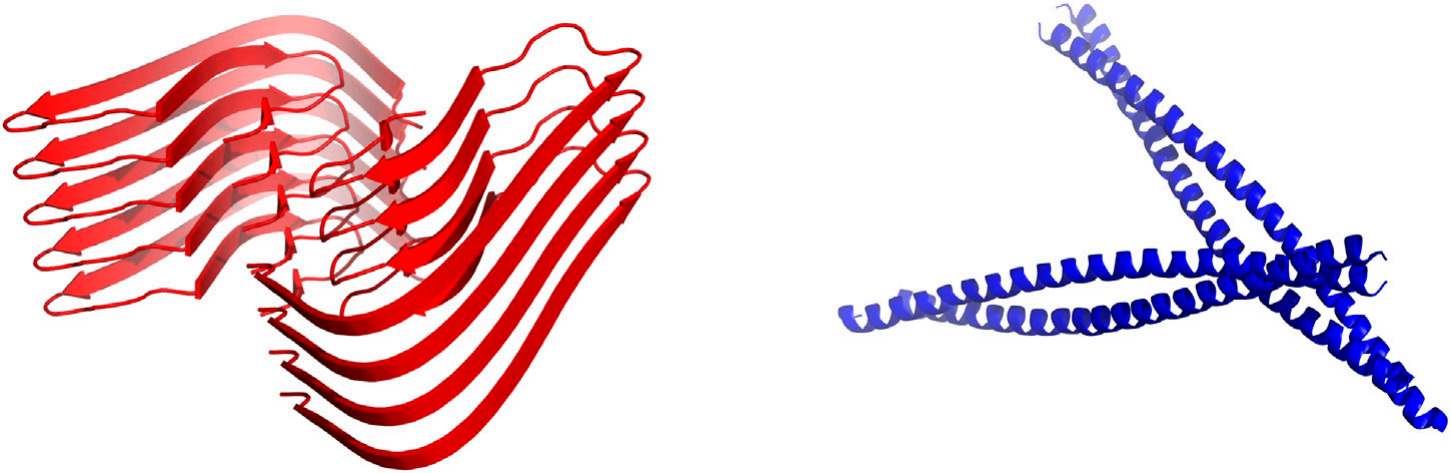
Structural building blocks of amyloid based and coiled-coil based IBs. Schematic representation of cross-β-sheet (red, according to PDB: 5OQV) and coiled-coil (blue, according to PDB: 2JEE) structures. They intend to illustrate the kind of scaffolds sustaining amyloid and coiled-coil based IBs.

A significant fraction of the aforementioned IB-tags promotes the formation of amyloid-based IBs (Cano-Garrido et al., 2013; Carrió et al., 2005; Choi et al., 2011; Morell et al., 2008; Wu et al., 2011) (Figure 1). In this supramolecular assembly, the amyloid architecture acts as a mechanically stable scaffold where the globular and functional protein is trapped (Cano-Garrido et al., 2013). However, these amyloid assemblies present two potential drawbacks: (I) A significant fraction of the target protein must be unfolded and inactivated to sustain the amyloid scaffold (Morell et al., 2008) and, (II) although IBs are assumed to be non-toxic entities, the release of toxic oligomeric species from the IBs cannot be entirely ruled out.

Different research groups have made use of the coiled-coil fold as a scaffold for the design of self-assembled protein nanostructures (Wu and Collier, 2017). The coiled-coil structure consists of two or more α-helices twisted around each other in a parallel or anti-parallel orientation (Figure 1). As conjectured by Crick in the 50’s (Crick, 1953), the sidechains of the residues from the different helices interlock along the core of the structure with a defined periodicity (heptad, hendecad, or pentadecad repeats), creating a distinctive packing named the “knobs-into-holes” (Lupas and Bassler, 2017; Lupas et al., 2017). We know now that the coiled-coil fold comprises a vast diversity of periodicities and structures, allowing to perform a wide range of functions in nature (Truebestein and Leonard, 2016) and opening the avenue for the design of bioinspired nanomaterials such as nanotubes (Xu et al., 2013), nanocages (Ljubetič et al., 2017) and vaccines (Morris et al., 2019).

In this context, the use of the coiled-coil motif as IB-tag might allow avoiding the caveats of β-sheet enriched IBs, since the assembly of these α-helical structures is not expected to impact the fold of the adjacent proteins or result in potentially toxic species. The first coiled-coil domain used to obtain functional IBs was the tetramerization domain of the cell surface protein tetrabrachion (TDoT) of *Staphylothermus marinus*, a right-handed and parallel tetramer consisting of an 11-residues repeat, which has been extensively exploited for building up catalytic IBs (Stetefeld et al., 2000; Diener et al., 2016; Kloss et al., 2018a, Kloss et al., 2018b; Jäger et al., 2018, Jäger et al., 2019; Lamm et al., 2020). Another coiled-coil domain employed to create catalytically-active IBs was 3HAMP, derived from the oxygen sensor protein Aer2 of *Pseudomonas aeruginosa* (Kloss et al., 2018a; Jäger et al., 2019). This domain consists of a homodimeric structure formed by two parallel monomers harboring three successive helical regions connected by flexible linkers (Airola et al., 2010). More recently, Garcia-Fruitós and coworkers reported using two leucine zippers (Jun and Fos) as IB-tags to produce fluorescent and antimicrobial IBs with remarkable specific activity (Roca-Pinilla et al., 2020a; Roca-Pinilla et al., 2020b). Without any doubt, these α-helical tags can be used to form active IBs; however, except for Jun and Fos, the biophysical properties of the IBs were not characterized in detail, and the preservation of the native α-helical structure in the final assembly could not be corroborated.

To provide a suitable coiled-coil tag that preserves its native structure when embedded in IBs, our group performed a computational analysis of the biophysical properties of different IB-tags and selected the ZapB protein, a non-essential factor involved in the cell division process of *E*. *coli* (Ebersbach et al., 2008), as an optimal candidate for obtaining coiled-coil inspired IBs. ZapB is a homodimeric and anti-parallel coiled-coil domain able to self-assemble *in vivo*, and therefore, it allows the production of α-helix-rich IBs when fused to a target protein, as demonstrated by circular dichroism and Fourier transformed infrared spectroscopy (Gil-Garcia et al., 2020). ZapB was fused to two different fluorescent proteins in our study, obtaining active and biocompatible nanoparticles displaying native-like fluorescent spectra and devoid of any amyloid character. As intended, these IBs were significantly more active than their amyloid-like IBs counterparts, indicating that the amount of inactivated target protein within ZapB-based IBs is low.

Although further biophysical studies of other coiled-coil-based IBs are needed to ascertain the generic non-amyloid character of these nanoparticles, the collected data suggest that this new class of IB-tags might substitute aggregation-prone sequences as a strategy for the production of high-quality functional IBs.

## Biomedical and Biotechnological Applications of Functional IBs: Is There Room for Improvement?

**FIGURE 2:**
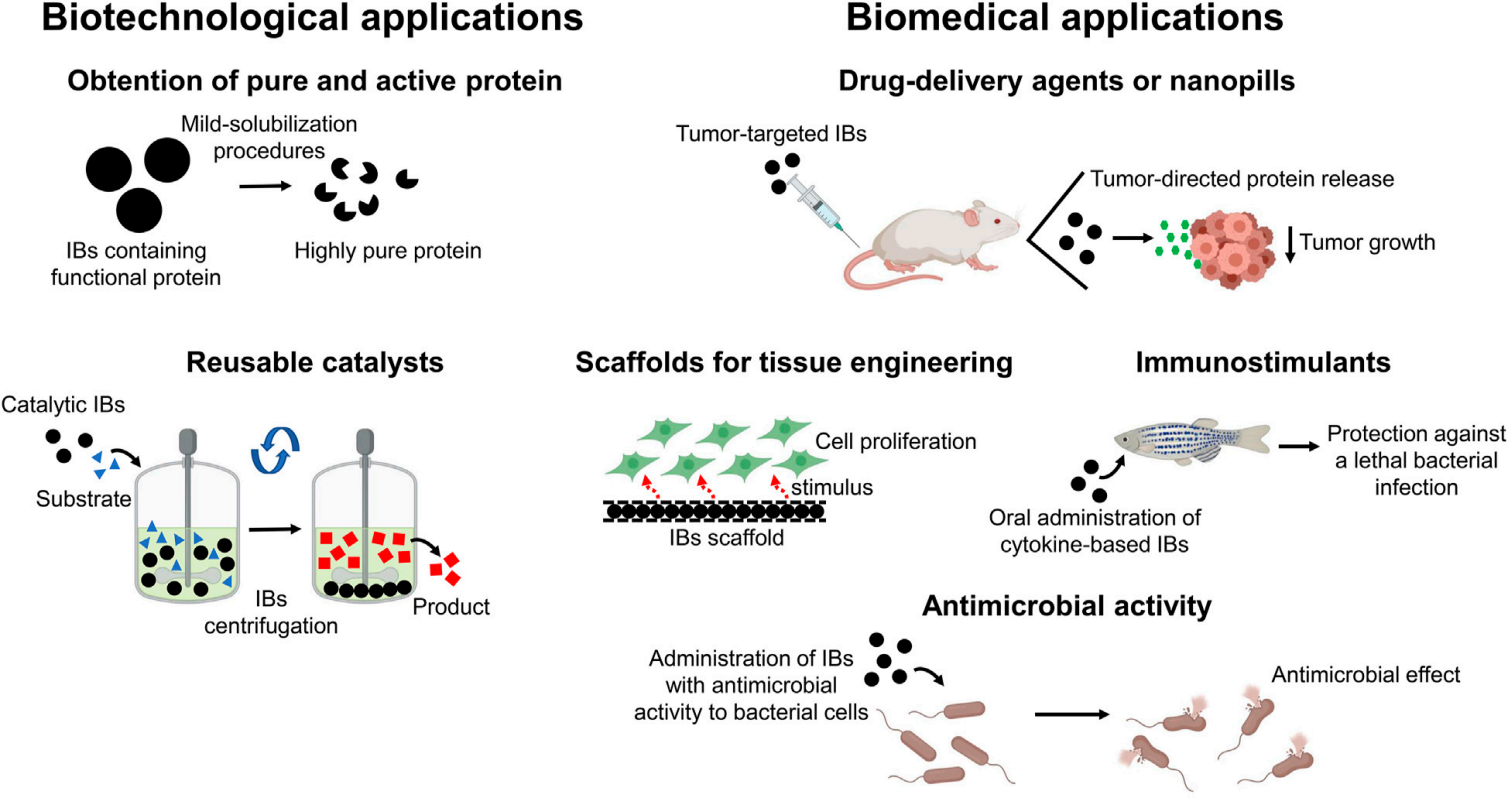
Biotechnological and biomedical applications of functional IBs. Functional IBs can be exploited for biotechnological purposes as a source of pure and active protein and as reusable and immobilized catalysts. Regarding their use in biomedical applications, IBs can act as targeted-nanopills, as biocompatible scaffolds for tissue engineering, as immunostimulants to act against lethal infections or as antimicrobial agents.

From a biotechnological point of view, IBs can be used as a source of pure and active protein, since the functional target protein can be easily isolated using mild-solubilization methods (for example, using low concentrations of organic solvents or alkaline pH) (Singh et al., 2015; Singhvi et al., 2020) (Figure 2). A more industry-oriented application of functional IBs is their use as reusable biocatalysts. Enzyme immobilization is a common strategy for improving the stability, enantioselectivity, and reusability of enzymes and the easy isolation of the reaction product (Mohamad et al., 2015). In this context, catalytic IBs fulfill the requirements of enzyme immobilization strategies, acting as porous and highly stable enzymatic reactors that can be separated from the reaction mixture by simple centrifugation (Figure 2). The use of catalytically active-IBs has been demonstrated for a wide range of different enzymes such as oxidases, reductases, and synthases, among others (Jäger et al., 2020), and different studies have reported striking recyclability for catalytic IBs (Koszagova et al., 2018), together with higher stability and activity under harsh conditions, like extreme pHs or the presence of organic solvents, than their soluble counterparts (Diener et al., 2016; Kloss et al., 2018a).

Apart from their biotechnological applications, IBs have been successfully applied in biomedicine acting as functional scaffolds in tissue engineering (García-Fruitós et al., 2010; Seras-Franzoso et al., 2013; Tatkiewicz et al., 2013), as targeted-delivery agents (Vázquez et al., 2012; Unzueta et al., 2018; Céspedes et al., 2019; Pesarrodona et al., 2019), or as immunostimulants (Schetters et al., 2020; Thwaite et al., 2018; Torrealba et al., 2016a, Torrealba et al., 2016b).

With regard to their application in tissue engineering, it has been demonstrated that the decoration of surfaces using IBs provides a mechanotransduction-mediated stimulation of cell adhesion and proliferation (via activation of the ERK pathway) (Seras-Franzoso et al., 2012) (Figure 2). This proliferative effect can also be achieved by the release of functional protein from IBs, as demonstrated for fibroblast growth factor-2 (Seras-Franzoso et al., 2014), with IB-decorated surfaces acting not only as a material to which cells can attach but also as proactive delivery agents. This property resembles that of the endocrine secretory granules, which store hormones in an amyloid-like conformation (Maji et al., 2009). Villaverde and coworkers have exploited this IBs property for delivering proteins to tumors, arresting their growth in mouse models of human colorectal (Céspedes et al., 2019) and breast (Pesarrodona et al., 2019) cancer (Figure 2). These IBs are constituted by a specific ligand for the CXCR4 receptor (overexpressed in metastatic cancer cells) and the PE24 toxin from *Pseudomonas aeruginosa*, or alternatively, by a specific ligand for the CD44 receptor and two anti-tumoral proteins, p31 and Omomyc.

Another intriguing biomedical application of functional IBs is the modulation of the immune system to fight lethal infections. IBs composed of cytokines such as TNFα displayed a prophylactic effect in zebrafish, protecting from infection by the pathogen *Pseudomonas aeruginosa*. This strategy overcomes the limitations associated with the low stability and short half-life of soluble cytokines (Torrealba et al., 2016a) (Figure 2).

Finally, the IBs technology can be exploited for the production of protein-based nanoparticles displaying antimicrobial activities. In this way, Roca-Pinilla et al. have been successful in the production of IBs composed of an antimicrobial polypeptide that combines different functional moieties, active against diverse Gram-negative and Gram-positive bacteria, including some multi-resistant strains (Roca-Pinilla et al., 2020b) (Figure 2).

The higher specific activity displayed by proteins when they are embedded in coiled-coil-based IBs allows forecasting that this type of assemblies would turn useful for biomedical applications, although their applicability in this context remains to be explored. First, the largest proportion of folded and active protein in these assemblies should permit reducing the critical concentrations for *in vivo* administration and the production costs. Additionally, lower doses would diminish undesired side effects, which, together with the lack of significant intrinsic toxicity demonstrated for coiled-coil based nanoparticles (Utterström et al., 2021), would make α-helix-rich IBs safer protein nanoparticles, mainly because they are expected to be devoid of potentially toxic β-sheet sustained conformations. Finally, because the native-like intermolecular interactions sustaining coiled-coil IBs are expected to be less robust than the strong amyloid-like contacts that glue β-sheet IBs (Carrió et al., 2005), these nanoparticles could display a higher release-efficiency upon their administration. However, an excessive release might be a drawback for their use as biocatalytic nanoparticles since it can negatively impact the product's purity and limit their recyclability.

## Conclusions and Perspectives

Initially considered waste products, IBs have opened as bio-nano-technologically relevant tools for a plethora of applications. The use of aggregation-inducing tags has allowed obtaining the desired target protein in an assembled state, independently of the protein identity, structure, or aggregation propensity.

In the last years, different strategies such as the use of lipopolysaccharide (LPS)-free bacterial strains have been implemented to create safe IBs devoid of bacterial toxic or immunocompromising components (Rueda et al., 2014; Gifre-Renom et al., 2018; Carratalá et al., 2021). An alternative to solve this problem is creating artificial IBs (ArtIBs) by assembling purified and initially soluble protein as IBs employing cell-free methods, such as the coordination of His-tags to divalent cations (Sánchez et al., 2020; Serna et al., 2020).

Considering the advantages of coiled-coil based-IBs and ArtIBs, we envision a promising avenue for the generation of a new class of functional IBs by mixing these two concepts since it has been demonstrated that the assembly of different coiled-coil proteins is dependent on the presence of divalent cations (Cristie-David and Marsh, 2019). In this way, a next generation of functional IBs can be potentially generated by creating ArtIBs assembled via coiled-coil interactions. On the one hand, the relatively high surface contact in this motif should render these nanoparticles highly stable, whereas the dependence on cations presence to keep their assembled state would allow tight and reversible control of their nanostructure.

All in all, the use of IBs as active nanoparticles is an emerging field that will continue cost-effectively providing new and unique applications, without the need for complex and harsh chemical reactions to assemble their active components; this environmentally friendly character is yet another advantage of this simple but effective technology.
